# Diversity of human and mouse homeobox gene expression in development and adult tissues

**DOI:** 10.1186/s12861-016-0140-y

**Published:** 2016-11-03

**Authors:** Thomas L. Dunwell, Peter W. H. Holland

**Affiliations:** Department of Zoology, University of Oxford, South Parks Road, Oxford, OX1 3PS UK

**Keywords:** Homeodomain, Embryo, Organs, Transcription factor

## Abstract

**Background:**

Homeobox genes encode a diverse set of transcription factors implicated in a vast range of biological processes including, but not limited to, embryonic cell fate specification and patterning. Although numerous studies report expression of particular sets of homeobox genes, a systematic analysis of the tissue specificity of homeobox genes is lacking.

**Results:**

Here we analyse publicly-available transcriptome data from human and mouse developmental stages, and adult human tissues, to identify groups of homeobox genes with similar expression patterns. We calculate expression profiles for 242 human and 278 mouse homeobox loci across a combination of 59 human and 12 mouse adult tissues, early and late developmental stages. This revealed 20 human homeobox genes with widespread expression, primarily from the TALE, CERS and ZF classes. Most homeobox genes, however, have greater tissue-specificity, allowing us to compile homeobox gene expression lists for neural tissues, immune tissues, reproductive and developmental samples, and for numerous organ systems. In mouse development, we propose four distinct phases of homeobox gene expression: oocyte to zygote; 2-cell; 4-cell to blastocyst; early to mid post-implantation. The final phase change is marked by expression of ANTP class genes. We also use these data to compare expression specificity between evolutionarily-based gene classes, revealing that ANTP, PRD, LIM and POU homeobox gene classes have highest tissue specificity while HNF, TALE, CUT and CERS are most widely expressed.

**Conclusions:**

The homeobox genes comprise a large superclass and their expression patterns are correspondingly diverse, although in a broad sense related to an evolutionarily-based classification. The ubiquitous expression of some genes suggests roles in general cellular processes; in contrast, most human homeobox genes have greater tissue specificity and we compile useful homeobox datasets for particular tissues, organs and developmental stages. The identification of a set of eutherian-specific homeobox genes peaking from human 8-cell to morula stages suggests co-option of new genes to new developmental roles in evolution.

**Electronic supplementary material:**

The online version of this article (doi:10.1186/s12861-016-0140-y) contains supplementary material, which is available to authorized users.

## Background

The homeobox gene superclass is large, with recent annotations indicating over 240 functional homeobox genes in human and over 270 in mice [1–3]. The large number of genes is mirrored by a vast range of reported expression sites and biological roles, such that few general statements can be made about homeobox gene function. Although the great majority of homeobox genes encode transcription factors, even this general statement might not be true for every gene since some homeodomains have reported roles in RNA-binding roles [4] or in modification of higher order chromatin structure [5]; a few vertebrate homeobox genes (CERS genes) even encode probable transmembrane proteins [6]. In biology, order can often be brought out of chaos if evolutionary history is considered. In recent years, we and others have attempted to build evolutionarily-based classifications of homeobox genes that should facilitate this [1, 7]. The classification of Holland et al. [1] divides the homeobox genes into 11 classes (ANTP, PRD, LIM, POU, HNF, SINE, TALE, CUT, PROS, ZF, CERS), subdivided into over 100 gene classes; the largest class can be divided into two subclasses (HOXL and NKL), although some genes are difficult to place, such as En and Dlx. The scheme of Bürglin and Affolter [7] is broadly similar but erects 16 classes, dividing PRD and TALE into two and five classes respectively.

The best known homeobox genes, such as Hox genes and some other ANTP class genes, have well-characterised spatial patterning roles in embryonic development, but there are also many reports of expression and function of Hox genes in adult tissues [8–10]. Other non-Hox homeobox genes, including many in the LIM class, can be considered to have more cell type-specific roles, rather than region-specific roles, in development and in adult tissues [11]. In contrast to region-specific or cell type-specific genes, more widespread expression might be expected for some homeobox genes, such as some in the TALE class, encoding co-factors of a range of homeodomain proteins [12]. At the extreme, the *POU2F1* gene has been reported as having ubiquitous expression [13]. Although an earlier study compared expression of all transcription factors [14], analysis at the level of homeobox gene family and class has not been undertaken; furthermore, much additional high-throughput expression data are now available. Hence, relationships between homeobox diversity and expression have not been tested.

We wished to investigate whether homeobox genes from certain evolutionary classes are expressed more broadly in adult tissues and organs than are genes from other homeobox classes. For example, we predict that ANTP and PRD genes are more restricted in expression than TALE and class genes, but is this prediction supported by data? Here we undertake this test, made possible due to the availability of a broad range of transcriptome sequencing (RNAseq) datasets, particularly from adult human organs. We also ask whether it is possible to establish sets of homeobox genes that are enriched in expression in particular datasets, providing ‘homeobox codes’ for adult tissues and organs.

Although human data are ideal for examining homeobox expression in adult organs because of the range of RNAseq datasets available, the same is not true for embryonic development. Several transcriptome datasets have been released for preimplantation human development [15–17], and we ask if there are sets of homeobox genes enriched at such early embryonic stages. To examine patterns after embryo implantation, mouse is a more amenable system and we test whether there are global changes to homeobox gene expression diversity during mouse development.

## Methods

To enable gene expression to be compared between tissues, organs and developmental stages, it is important to calculate expression levels using identical methods for each RNAseq dataset. To enable this, we did not use published FPKM data (fragments per kilobase per million sequencing reads) or RPKM data (reads per kilobase per million sequencing reads), but took publicly-available RNAseq data files for each human tissue, organ sample or developmental stage, and remapped the raw sequence reads to human genome assembly NCBI GRCh38.p2. For most tissues, organs and developmental stages, replicate RNAseq datasets were merged (Additional file 1: Table S1). We used the STAR RNA-seq aligner [18] using the default settings with the addition of --outSAMstrandField intronMotif and --outFilterMultimapNmax 15 for mouse and 30 for human to increase the limit for multimapping reads before they would be discarded; this improves accuracy of expression analysis from repeated loci.

For human data, we used a collection of 331 SRA datasets analysed previously [19]. These comprise 5850 million paired end sequence reads and 3376 million single end sequence reads representing 59 developmental stages or tissue types. Read mapping to most homeobox genes was performed in [19]; to this analysis we added *NANOGNB*, *CPHX1* and *CPHX2* (Additional file 2: Figure S1). This analysis gave FPKM (fragments per kilobase per million reads) data for 242 human homeobox genes comprising all human loci listed by Zhong and Holland [2] after excluding 90 pseudogenes and several closely similar duplicated Dux loci. LOC647589 has been named *ANHX* (*Anomalous homeobox*) gene by PWHH, Elspeth Bruford and Ying-fu Zhong (www.genenames.org). To avoid spurious or background read counts conflating analysis, we considered any FPKM value <2 as equal to zero. Classification of human homeobox genes followed Zhong and Holland [2], based on Holland et al. [1], except that *CPHX1* and *CPHX2* are here placed in the PRD class following Töhönen et al. [17]. *NANOGNB* is here provisionally considered in the ANTP class on the basis of chromosomal location, *TPRX2* is considered a functional gene rather than a pseudogene [19, 20], and DUX loci are restricted to *DUXA*, *DUXB* and *DUX4.*

Identical methods were used for mouse homeobox genes and RNAseq data sets, using genome assembly GRCm38.4. In total, 298.4 million single end sequence reads and 983.1 million paired end reads from 71 SRA datasets representing 12 developmental stages were mapped (Additional file 1: Table S1). Data are reported as derived from whole embryos. The mouse homeobox gene set comprised 278 genes and followed Zhong and Holland [2], with the exception of some minor annotation differences within the complex Obox, Crxos and Rhox3 gene families. Classification of mouse homeobox genes followed Zhong and Holland [2], except that *Cphx*, Gm2104 and Gm2135 (now renamed *Cphx1*, *Cphx2* and *Cphx3*; http://www.informatics.jax.org/) were placed in the PRD class, along with *Crxos1*.

## Results and discussion

### Diversity of homeobox gene expression in human tissues and organs

**Fig. 1:**
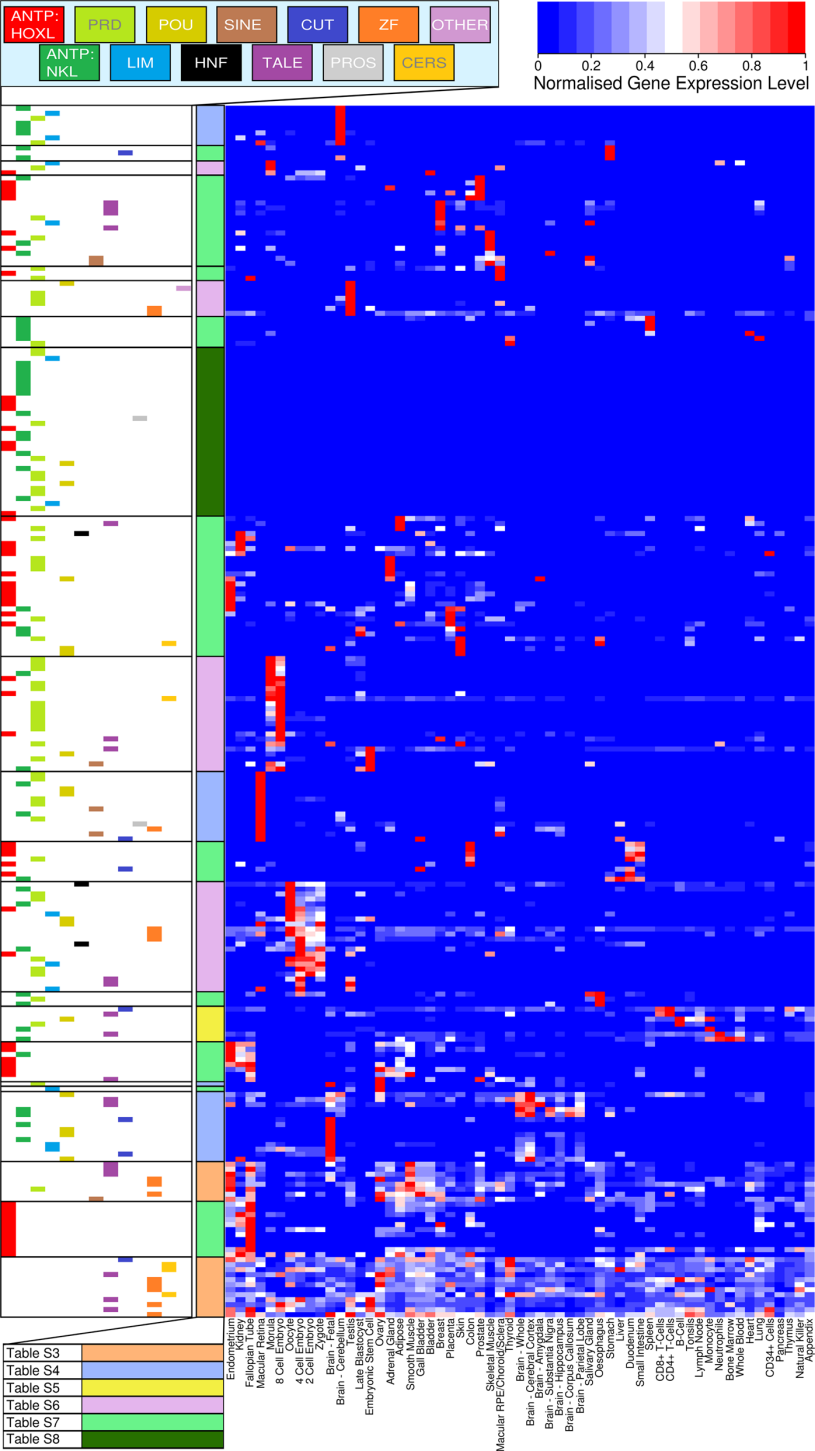
Heatmap showing human homeobox gene expression. Expression data for 242 homeobox loci across 59 human cell types and tissues clustered according to expression level after normalising individually to the maximal expression of each gene. Red high expression; blue low or zero expression. The *horizontal coloured bars* indicate the homeobox class for each gene. The same figure, showing gene names, is given in Additional file 4: Figure S2. The colour coding of each larger grouping corresponds to Additional file 5: Tables S3 to S8 where genes in each group are listed

To assess which tissues and organs express each homeobox gene, we mapped publicly available RNAseq data to the human genome and calculated FPKM values for every homeobox gene (Additional file 3: Table S2). Figure 1 shows relative gene expression levels (normalised to maximum expression for each gene), clustered according to expression profile (Additional file 4: Figure S2 shows the same, but with gene names). From this analysis, we compiled lists of homeobox genes with similar expression profiles across adult human tissues or preimplantation stages (Additional file 5: Tables S3–S8).

A clear pattern is that most homeobox genes have moderately specific expression patterns; by this we mean that most genes have one site of maximal expression (shaded in red in Fig. 1), and few other tissues with high or moderate expression, with most tissues being negative or substantially lower. There are important exceptions, however, and we identify 20 homeobox genes with very widespread expression profiles across a large number of tissues (peach coloured categories in Fig. 1 and Additional file 4: Figure S2; listed in Additional file 5: Table S3). These widely-expressed genes include six TALE class genes, including several genes (*MEIS1, MEIS2, PBX1, PBX3*) whose protein products are known to form co-factor complexes with a range of partner transcription factors [21]. This role as common co-factors may explain the widespread expression we detect. Also included in the list of widely expressed homeobox genes are *PRRX* (PRD class), *SIX5* (SINE class), *CUX1* (CUT class), three members of the CERS class encoding transmembrane proteins, and eight members of the ZF class. We propose that these genes have general roles in cellular functioning. It is perhaps surprising that *POU2F1* is not among the list, since this gene has formerly been described as ubiquitously expressed [22]. The reason is that elevated expression in preimplantation stages causes this gene to cluster with preimplantation-specific homeobox genes. It is striking that there are no ANTP class genes in the ‘widespread expression’ category, despite these comprising the largest homeobox class in humans (101/242 genes in the current analysis). This finding further supports the contention that ANTP class genes are primarily involved in spatial patterning during embryonic development.

Additional file 5: Tables S4 to S7 list sets of homeobox genes that show degrees of tissue specificity; these groupings are generated by expression clustering analysis. Biologically similar tissues, such as ‘neural tissues’ or ‘immune-related tissues’, form distinct groups in the analysis. Additional file 5: Table S4 (blue in Fig. 1 and Additional file 4: Figure S2) comprises genes expressed predominantly in brain and neural tissues, including cerebral cortex, corpus callosum, hippocampus, parietal lobe, amygdala, substantia nigra, foetal brain and tissues of the eye. Different homeobox genes are expressed in distinct subsets of these tissues, as shown in Additional file 5: Table S4. There are no Hox genes in this set, despite the fact that numerous studies have examined the role of Hox genes in neural patterning. However, we note that the neural RNAseq data analysed are derived predominantly from anterior brain regions whereas most studies of vertebrate Hox gene expression reveal spatial expression only in body regions posterior to the middle of the hindbrain [23]. Adult forebrain expression of Hox genes has been reported [10] but is relatively low level, explaining why this does not show as a major Hox gene expression site in our analysis. Even though Hox genes do not feature in the ‘neural-enriched’ set, it does include several other ANTP class genes including several implicated in specification and patterning of anterior brain regions in other vertebrates: *BARHL1, BARHL2, EN1, EN2, TLX3, NKX6-2, NKX2-2, DLX1, DLX2, HMX1, VAX2, GSX2*. Amongst the PRD class, homeobox genes in this dataset include the retinal gene *CRX*, the *PAX6* gene which is mutated in aniridia, two human Rax genes and the two human Vsx genes.

Additional file 5: Table S5 (yellow in Fig. 1 and Additional file 4: Figure S2) includes homeobox genes predominantly expressed in immune tissues such as B-cells, T-cells, monocytes, neutrophils and bone marrow. These include several homeobox genes known to be associated with immune function notably: *PAX5*, somatic and germline mutations in which are associated with B-cell precursor acute lymphoblastic leukemia [24]; *HLX* which modulates interferon expression in T-cells [25]; *SATB1*, encoding a chromatin loop-associated homeodomain protein implicated in T-cell development [7]; *POU2F2* required for B-cell maturation and survival [26]; *VENTX* involved in macrophage differentiation [27]. The inclusion of *PBX2* and *PBX4* in this set is more surprising and suggests further investigation. We caution, however, that the precise delineation of the ‘immune-enriched’ dataset (unlike most other tissue datasets) is sensitive to changing the FPKM cut-off used for defining expression versus background (not shown).

Additional file 5: Table S6 (pink in Fig. 1 and Additional file 4: Figure S2) comprises homeobox genes expressed predominantly in reproductive tissues and early development, specifically testis, placenta, oocyte and preimplantation embryos (zygote, 2-cell, 4-cell, 8-cell, morula, blastocyst). Several homeobox genes have already been described as characteristic of one or more of these tissues or developmental stages, and these are found in our list. Examples include *NANOG* and *POU5F1* which are well-characterised markers of pluripotent cells and several totipotent-cell expressed PRD class genes that have been the focus of recent functional studies (*ARGFX, CPHX1, CPHX2, DPRX, LEUTX, TPRX1, TPRX2, OTX1, OTX2*) [19, 20]. Interestingly, many other homeobox genes also cluster in this set on the basis of their expression, including two Hox genes (*HOXD1, HOXC13*) indicating they are worthy of further study in this regard (Additional file 5: Table S6). *Hoxd1* expression has been previously reported in preimplantation mouse and cow embryos [28–30] but not to our knowledge *Hoxc13*; however, one of the two *hoxc13* duplicates in zebrafish is expressed in early cleavage stages [31].

**Fig. 2:**
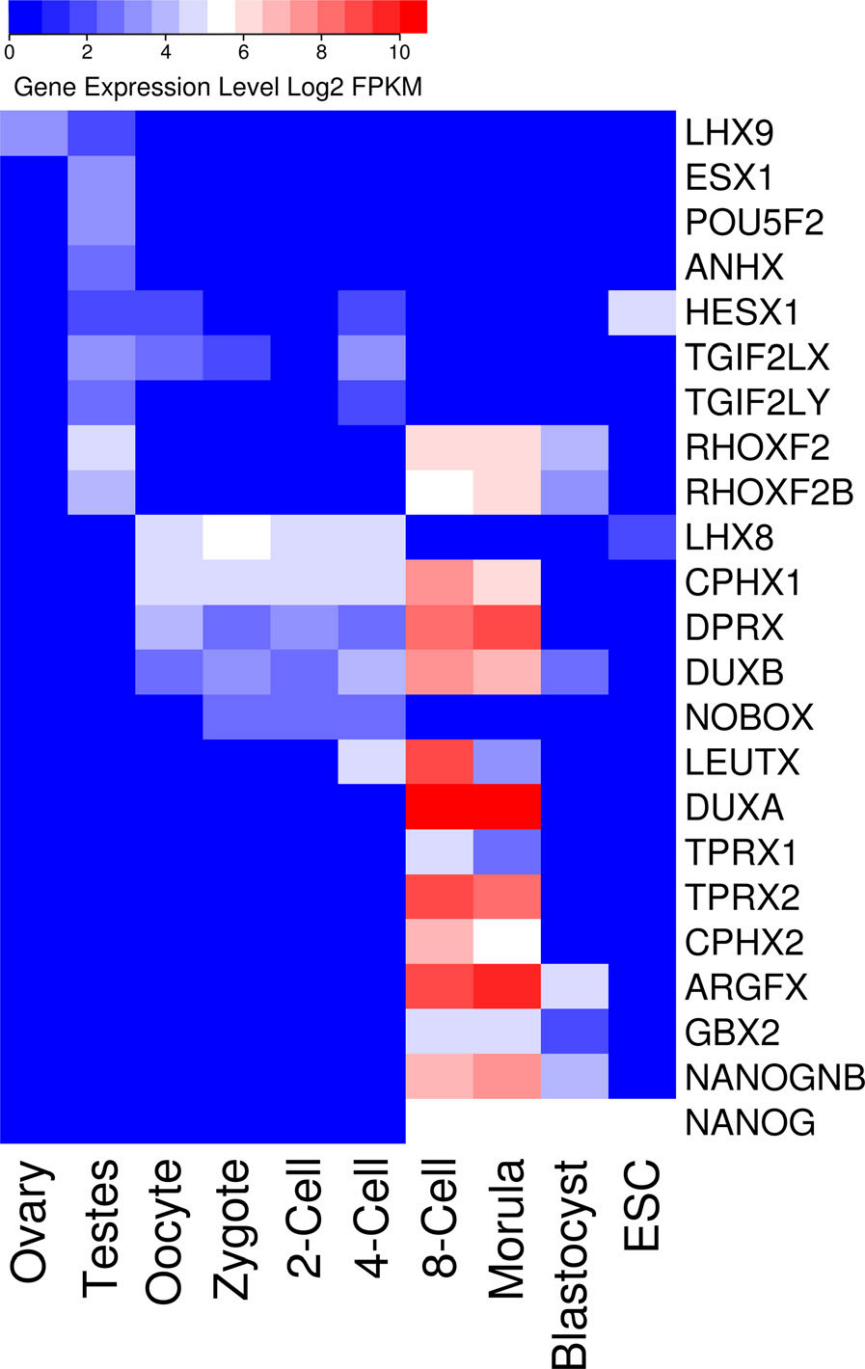
Heatmap showing gene expression for human homeobox genes expressed specifically in reproductive tissues, development stages and embryonic stem cells. A gene was determined to be ‘embryo or reproductive tissue-specific’ if the FPKM expression level was greater than 2 in one or more stages and below 2 in all examined adult tissues

We refined the analysis to identify homeobox genes that are expressed only in these reproductive tissues and developmental stages (no expression > =2 FPKM in other cell types or tissues); we also added ovary to this set, as this was not grouped with them by expression clustering methodology. We identify 23 human homeobox genes that are expressed exclusively in reproductive or very early developmental tissues in this analysis (Fig. 2). Over half (13/23) have a clearly defined maximum expression level confined to a small developmental window from 8-cell to the morula stage of embryo development. Not only is the expression of these genes tightly regulated, but we note 12 of them (*RHOXF2, RHOXF2B, CPHX1, CPHX2, DPRX, LEUTX, TPRX1, TPRX2, ARGFX, NANOGNB, DUXA, DUXB*) are phylogenetically restricted to within eutherian mammals [19, 32–34]. The correlation between tight expression specificity and similar phylogenetic distribution suggests there may have been selective pressures to co-opt novel homeobox genes to new developmental roles during the evolution of eutherian mammals. The peak of 8-cell to morula suggests these genes may combine to prepare the totipotent stages of embryonic development for subsequent cell fate specialisation. Indeed, two recent studies have postulated regulatory roles for several of these genes during early human embryo development [19, 20].

Additional file 5: Table S7 (light green in Fig. 1 and Additional file 4: Figure S2) lists an assemblage of homeobox genes with predominant expression in particular organs system; these organs do not necessarily group together in expression clustering. For example, two genes have highest expression in gall bladder (*ONECUT1*, *ONECUT2*), several posterior Hox genes plus *EVX1* and *NKX3-1* associate with colon and prostate, and *PDX1* is in duodenum. Other examples are given in Additional file 5: Table S7.

Additional file 5: Table S8 (dark green in Fig. 1 and Additional file 4: Figure S2) groups homeobox genes that do not have clear expression in the RNAseq datasets under study. Many of these are genes with well characterised roles in mid to late embryonic development in other vertebrates (e.g. *CDX4, EVX2, GSX1, DMBX1, PAX4, PAX7*); it is likely that their assignment to this category reflects the fact that in this analysis we used adult human tissues and preimplantation stages since there are few RNAseq datasets from postimplantation human development; model species such as mouse are more amenable for studying such developmental stages.

### Homeobox genes expressed in mouse development

**Fig. 3:**
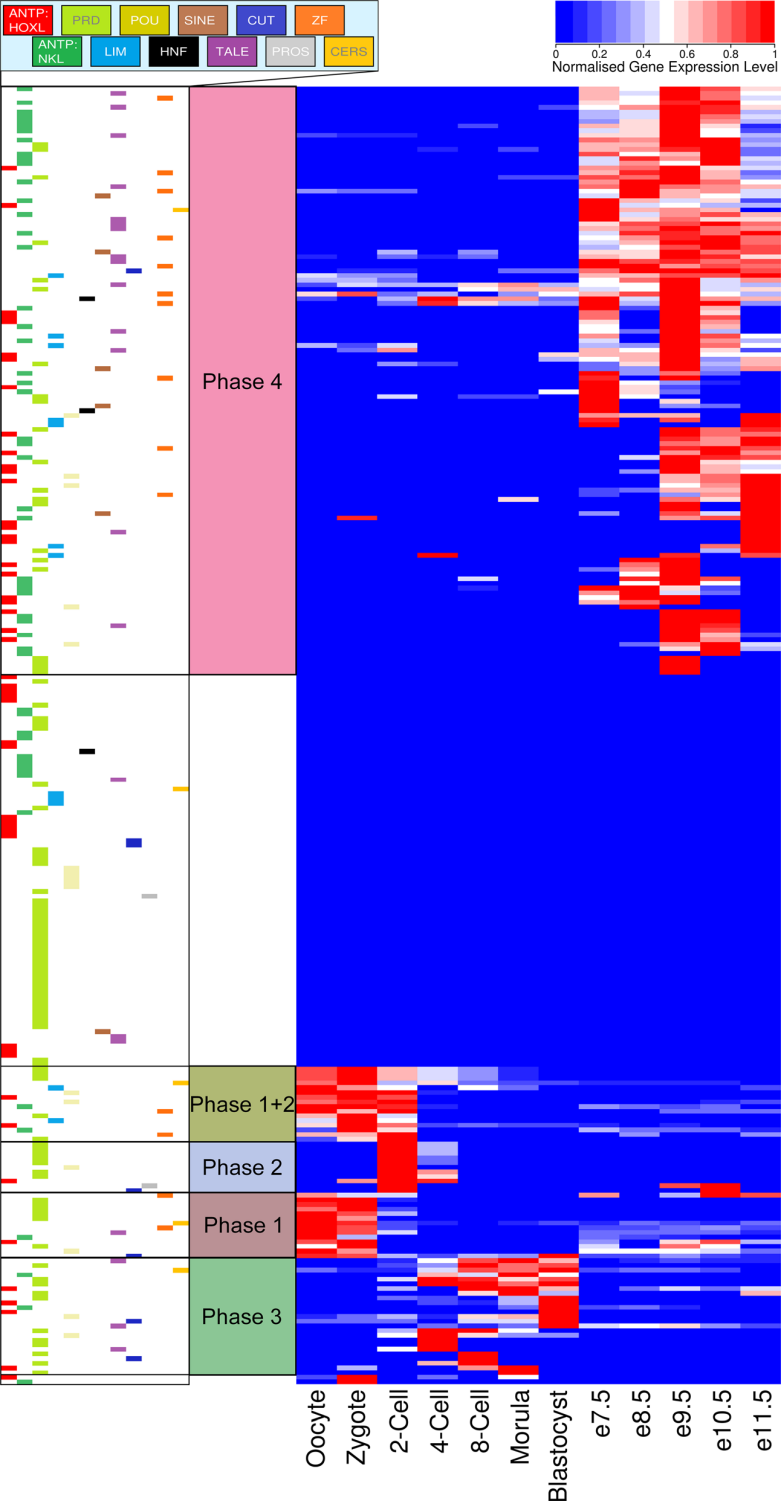
Heatmap showing mouse homeobox gene expression in development. Expression data for 278 mouse homeobox loci across 12 embryonic stages clustered according to expression level after normalising individually to the maximal expression of each gene. *The horizontal coloured bars* indicate the homeobox class for each gene. The same figure, showing gene names, is given in Additional file 6: Figure S3. Genes in each temporal group are also listed in Additional file 7: Table S9

To examine temporal patterns of homeobox gene expression through postimplantation mammalian embryonic development, mouse is a tractable system. We mapped publicly available RNAseq data to the mouse genome and calculated FPKM values for every homeobox gene (Additional file 3: Table S2). The datasets analysed ranged from oocyte to 11.5 days’ post coitum (e11.5); we excluded developmental stages later than e11.5 as most major organs systems are forming by e12.5, meaning that total embryo datasets become complex amalgamations of parts. There is also a temporal gap between blastocyst (~e4.5) and e7.5, which reflects the practical difficulties of identifying and dissecting embryos at the earliest post-implantation stages. Figure 3 shows relative expression levels (normalised to maximum for each gene), clustered according to expression profile.

The most striking features of this analysis are several clear temporal shifts in the clustered patterns of gene activity, which could be described as four ‘phases’ of gene expression separated by ‘gear changes’ (Fig. 3). Gene names are given in Additional file 6: Figure S3 and Additional file 7: Table S9. First, from oocyte to zygote, a set of maternal transcripts predominate, with these genes showing little expression later than this stage. These transcripts derive predominantly from homeobox genes in the PRD class. Second, at the 2-cell stage, corresponding to the first stage of embryonic genome activation (EGA), a clear and distinct set of PRD class genes is activated; few of these persist to the 4-cell stage. These genes include homologues of the human PRD genes, noted above, that are expressed in human from 4-cell or 8-cell to morula (Fig. 2). One group of homeobox genes, from multiple classes, spans phases 1 and 2 in their profile of expression. Third, at the 4-cell to 8-cell stage another distinct set of homeobox genes is activated, with many of these genes persisting in expression until blastocyst. Fourth, the expression profile from e7.5 onwards is strikingly different, although we are missing the fine temporal detail of the transitions between blastocyst and e7.5. Thus, there is a very clear distinction between the homeobox genes expressed in preimplantation stages, and the homeobox genes expressed in post-implantation stages (Fig. 3 and Additional file 6: Figure S3). Within the latter group there is considerable variation, with some genes initiating strong expression at e7.5 and others as late as e9.5. The group of genes that initiate as late as e9.5 is dominated by members of the HOXL subclass of the ANTP class, including many canonical Hox genes. We suggest this increased deployment of the ANTP class marks the principal phase of spatial patterning, as positional identities are conferred to regions along the anteroposterior body axis within each germ layer and incipient organs are specified.

### Do classes of homeobox gene differ in tissue specificity?

**Fig. 4:**
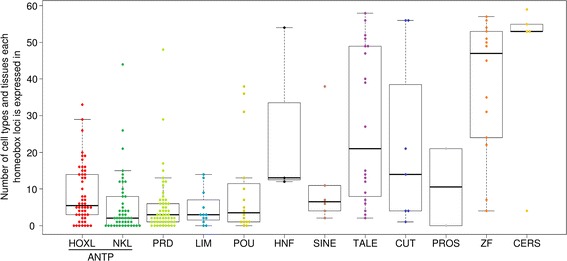
The number of human cell types and tissues in which individual human homeobox loci are expressed. Each *coloured dot* indicates an individual homeobox locus. Boxplots were generated using boxplot function in R; the box defines the 25^th^ to 75^th^ quartiles with the 50^th^ identified by the *horizontal line*, whiskers define limits outside which samples are classed as outliers

The same gene expression data were used to examine whether different classes of human homeobox genes have more or less tissue specificity. We find considerable spread of expression specificity within each of the 11 homeobox gene classes (Fig. 4). Four classes show high tissue specificity (a small range of tissues expressing): ANTP, PRD, LIM and POU. The high specificity is particularly striking for ANTP and PRD classes, as these contain large numbers of genes (101 and 55 in this analysis). This high specificity is consistent with roles in cell-type and tissue-type specification, and also for regional patterning if subsequent roles are predominantly located within organs developing within restricted spatial domains. It is notable that ANTP, PRD, LIM and POU classes (and the SINE class) have been considered metazoan-specific [35], consistent with a model in which homeobox genes were recruited for spatial patterning specifically in metazoan evolution. In contrast, the HNF, TALE, ZF and CERS homeobox gene classes show less tissue specificity, although individual genes reveal exceptions to the pattern and ranges overlap. This finding supports our prediction that the TALE class would show less specificity than the ANTP and PRD classes; the findings for ZF and CERS are revealing and suggest widespread roles for most homeobox genes within these classes. One possible source of artefact for this analysis would be if different tissues themselves expressed radically different numbers of homeobox genes; if this were the case they could not be treated as equivalent datasets. However, although there is variation between tissues this is not extreme, with most human tissues in the dataset expressing between 25 and 65 homeobox genes (Additional file 8: Figure S4).

## Conclusions

We have examined the tissue specificity of gene expression across the homeobox gene superclass of humans, and the temporal profiles of expression for homeobox genes of human and mouse. Several key findings emerge from these analyses.

First, we identify a set of 20 human genes with very widespread expression, including multiple members of the TALE, CERS and ZF classes, and single members of the PRD, SINE and CUT classes. We suggest these genes have general roles in cellular functioning.

Second, most homeobox genes have relatively distinct tissue specific expression, and we compile and present distinct lists of human homeobox genes with enriched expression in neural tissues, in immune tissues, in reproductive and developmental samples, and in numerous organ systems.

Third, we have identified 12 eutherian-specific homeobox genes with strikingly specific expression patterns during the 8-cell and morula stages of human embryo development. The expression of these genes is not detectable outside of reproductive tissues or the embryo.

Fourth, we identify four distinct phases of homeobox gene expression in mouse development, specifically: oocyte to zygote; 2-cell; 4-cell to blastocyst; early to mid post-implantation. The most dramatic shifts in homeobox gene expression are between 2-cell and 4-cell, and between blastocyst and post-implantation. Within this group there is a gradual shift in expression between e8.5 and e9.5 dominated by new expression of HOXL ANTP class genes.

Fifth, we find that distinct classes of homeobox gene differ greatly in specificity of expression: ANTP, PRD, LIM and POU have highest tissue specificity; HNF, TALE, ZF and CERS are the most widely expressed.

## Additional files

Additional file 1: Table S1. Human and mouse SRA data file accessions. (XLSX 30 kb)
Additional file 2: Figure S1. Human coding sequences. Deduced coding regions of human *NANOGNB, CPHX1* and *CPHX2* genes using human embryo RNASeq data. (DOCX 13 kb)
Additional file 3: Table S2. Raw and normalised FPKM expression values for human and mouse homeobox loci for all analysed cell types and tissues. (XLSX 265 kb)
Additional file 4: Figure S2. Heatmap showing human homeobox gene expression and gene names. Expression data for 242 homeobox loci across 59 human cell types and tissues clustered according to expression level after normalising individually to the maximal expression of each gene. Red high expression; blue low or zero expression. The horizontal coloured bars indicate the homeobox class for each gene. (PDF 271 kb)
Additional file 5: Tables S3–S8. Gene names for human homeobox loci identified in major expression clusters. (XLSX 51 kb)
Additional file 6: Figure S3. Heatmap showing mouse homeobox gene expression in development and gene names. Expression data for 278 mouse homeobox loci across 12 embryonic stages clustered according to expression level after normalising individually to the maximal expression of each gene. The horizontal coloured bars indicate the homeobox class for each gene. (PDF 225 kb)
Additional file 7: Table S9. Gene names for homeobox loci expressed in four major expression ‘phases’ identified during mouse development. (XLSX 18 kb)
Additional file 8: Figure S4. The number of homeobox loci expressed in each of the assessed tissues and cell types. An FPKM of 2 was used as a cut off above which a loci was classed as expressed. Homeobox loci are separated by expression level for each cell type or tissue. (PDF 143 kb)

## Abbreviations

FPKM: Fragments per kilobase per million sequencing reads
RNAseq: RNA sequencing
RPKM: Reads per kilobase per million sequencing reads
