# Supplementary material for: Diversity of human and mouse homeobox gene expression in development and adult tissues
Source: BMC Dev Biol. 2016 Nov 3;16:40. doi: 10.1186/s12861-016-0140-y (PMC5094009; doi:10.1186/s12861-016-0140-y)
Supplement: Additional file 2: Figure S1. — Human coding sequences. Deduced coding regions of human NANOGNB, CPHX1 and CPHX2 genes using human embryo RNASeq data. (DOCX 13 kb) [file 12861_2016_140_MOESM2_ESM.docx]

NANOGNB

Exon 1 and UTR

GGGATTTAACCTTGAGTGTGATTGAAAAAGCACAGGGACAGAAATCAGTAGGGGAAAGAGGAGCAACAAAGAAAGCTTTGAAAGAGCAGCCATTGACTTGAGGAAGCCCCAGGAAAGGATGGTGTTCAGGAAGTGGGAGAATAAACTCTTTCAATGTGGAGAGAGATCCCGATACTTCAGAAGCCCCTGATCATTCAA

Exon 2

AAACAATCAGCTATGCCTTGGGATCAAGATCCAGAACAATCAACTGGAAATTACAGTGAAGATGAACAAAATGGAAAGCAGAAATGGAGAGAAGAAGGAGAAGCAGGCAGAAAGAGAGAACGAGAAAAAGAAGAAAAAAACGAAAAGGAGCTGCAAGATGAACAGGAAAACAAAAGGAAAAGGGAAAATGAGAAACAGAAACAGTATCCCGAGAAAAGATTAGTCAGCAAATCCCTCATGCATACTCTCTGGGCAAAGTTTAAGTTAAACAGGTGCCCCACTATACAAGAGAGTCTATCACTGTCATTTGAATTTGACATGACACATAAACAG

Exon 3

ATAAGTCAATGGTTTTGTAAAACGAGGAAGAAATATAATAAAGAAATGTCCAAGAGAAAGCATAAGAAAAAACATATGAG

Exon 4 and UTR

ATGGAGATCTCTGTGTTGCCAAGGCTGGTCTCGAACTCCTGCCCTCAAGTGATCTTCCTATTTTGGCCTCCAGAAATGCTGTGATTACAAGCATGAGCCATCGCACTGGCTAAGACATTTTACATGACACCATTCTCACCAATAAATGGAGTTCTGAAAGGATAAACAAGAAAACATT

CPHX1

Exon 1

CTCAGTTGCTTGCTGATCTCAGTTGCTTGCTGGTCTCCGTGAAGTCCAGGTCTTCTACCCCAAGATGAATTTGGACGGCACTTCAGGTG

Exon 2

GTTTCCCAGCTGAAGAGGATCATCATAATGAAGAAAGACAAACAAAGAATAAAAGAAAAACAAAACACCGACATAAATTTTCTGAAGAATTACTGCAGGAACTTAAGGAAATATTTGGAGAGAACTGTTATCCCGATTACACAACTAGGAAAACACTGGCCATCAAATTTGATTGTCCGGTAAATGTGATAGAT

Exon 3

AATTGGTTCCAGAATAAAAGAGCCAGACTTCCACCTGCAGAAAGACGCAGAATATTTGTTCTTCAGAAAAAGCATGATTTTCCAGTCCAAGCCCATTCATTTTTAAGCTGCCAGGAGACCCAGGCTGCAGCTCACAACTATGCCACCAAGCAGAGCCTCTCTGGTGCCCAGAGGGCTCTGATGAGAAGAGCTGGTTGCTCCCATCTGGAGAAACAGTGGATTCCCAGTCAAGAAATGGGCTATAATTGTTTCTCTTTGGAGAACCAAGAGACTCCCAGTCAACAGGTGGGCCCCCAGTGCTCTTATCTGGAGAAACCAGGGATTCCCAGTCAACAGGTGGGTTCCCAGTGCTCCTATCTGGAGAAACTAGGGATTCCCAGTCAACAGGTGGCCTCCCAGAGTTCCTATCTGGTCACAGGCACTGAAAAGCATCCAGGCTGTGCTATGGGGTATGGAGGTGACACAGGAAGTGGGCATTCTGGAAGTGGGCATTCTACTGCCTATCATTTTCTCAGCTACAACTCTGCAGAATGCCTTCATCCTCCCCCATCTTCTGTGCCATATTTTCATGGAGAAAGGACTGAAACCAAGGAAAGCCAGCATGCAAGTCCTTTCCTTTTGGATTACGCTCAAGGTGCATATGGGGTGAAGAAAGACCATTGTCTTTGCTCATTCTGTCTCTCACTGCTGGGACAACAGCAGCAGAATGATTGGCAGTATCACCTGCAGCAGCACCAACAGCCTCAGAATTACTTAGAGGGGATGATGTTGCAGGAACAGCTCCCAATGGACTCGGGTCCTTGGGATCTAGGGAAGCAGTGGTCCTCGGCTCAGTCACAGCTGCAGAGTCAACTGCCTCAGAATAATGGAAAGCCGTTGTGCTCTCAACTGCAGCACATGTCTCTCCAAATAGCTGCCGACTCACCCCTTCTGCCTCTGGGGCAAGATATGCAGGAAAGGGCTTCAGAGCAACCCAGGACCCAAATGCAGCAACTTTAAGTTGAAGAGTGGACAAGGGTCTGCAGCAGGGAACCAAGGATGCAACTCTGCAAAGTAGTGCTAAGTGTCAGAGAAACACAGTCGCAGGTCAGGCCCACAGTCACCCACACAGTAGCACAAGGCAGAGCCCTTACCAGAAGCCTCCACATTAAGAAAAGAAAGAAAGAAAGAAAGAAAAAAGCCAGGTGACATTGTTTGAGCCCAGGAGTTCAAGAAAAGCCTGGGCAACATGGTGAGTCCCTGTCTCTATAAAAAAACTTTTTAAAAAATTAGCTGGGCATGATGGTGTGCTCCTGTGGTCCCAGCTACTCAGGAGGCTGAGGTGGGAGGATCACTTGAGCCCAGCAGTTTGAGACCAGCCAGGGCAATATTGGAAGATCCCATCTCTACAGAAAAATAAAAAAAAATAAAAAATTTAGCCAGGCATAGTGGCTTGTGCCTATGGTCCCAGCTACTTCAGAGGCTGAAGTGAAAGGATCACTTGAACCTCAGAGGTGGAGGCTGCAGTGAGCCGTGATCATTCCACTACACTCCAGCCGGGGTGACAGAGTAAGACCCTGTCTATGAAAAATTAAAAAGAAGAAGAAAAATAAGACTGTCTACAGGAAGGCTCCATGAGCTCTTGGTCCCTTCTCATACAGAAGCGGTGTCCACATGTGTTACAGCTCCTGAGCTCCTGACCATATAGTTTACAGTGGCCTTGAAAAGACTGACTCCATAGAATATGGATTGAAAAACCAACGAGCACAAAGATTGCAGGAGCACTGCTCAAATCACATCATGCAAAGTCTAATGACATTTTGCCTTCAGAAGAAGGAGGCTCTTGCCCAGCCCTTCAGGCATTGTTCCCCTGAGAGGCAATATAGCCAACAAGAACAGAGTAACCACAAAGCCCCACAGCAGTGACAATTGGATGGGGGATTCTAAAGGCTACTCTTGCAAAACTCTCTCCTCCATGGACCAGAGCAGTATCTGCAGGTTGTGAAGCGTGGCCTACCAGGACCCAGCTGTATCTCCTCAGCAGCCAGCCCCACCGAAGAAGCTCTGCCTGTGAAGGGTGCTGCAGCAACATCAGCCTGGCCTTTGCACATCTCAAAGTCAGCCCAGTATAAAGGCAGGAGCTTTTAATTGAAAGGCATTTAAAGACACAGTACATAAAGCCAGGCACAATGGCTCACACCTGTAATCTTAGCACTTTGGGAAGCCAGGGCAGGAAGATCTCTCTGAGCCCAGGGATTCAAGACCAGCCTGGGTACCATGGCAAGATCTTGTCTCTAGAAAAAGGAAGAAAAAAAAAACCATGCAAGAGGACCTGAAAATCCTCAGGAAGATGCTGTGCTGAAGCAGCCCCCACCAGCCCTCTTAGCTGACAGCTGCTGCCCAGGAATCCAGAGTGTTGTGACCAAGTCATCTAGGGATGTGATGCCAAGGCCAACAAACATCTGAAATACCCAG

Exon 4

AATTTGCTGATAAACTCAATGAGCAAAAACTTGTCCAACTAGAAGAGGCGAAGCAGGCTTCCATCAAACAAATCCAGGATGCAACTGATTTGGAGAAGTCACAGCAG

Exon 5

TTTCAACATAACAACTGATGAGCTTTTCTTCCTGATGAGAGACCACCAACCATGGAATGATTCTGGCCAGTCTATGACGAATGCCCAGTGAGGGTTTTTGTGTCTCTACTTCACCTTTGGATGTCAAAGAGCACAACACTCCACCCTGGGATCACGCTGATGCCGCCGTTTTTTGAACGTGGGTCCTACGGAGAGGCATGAAGCTCAATTGCATATATATTTGTTTCTCTTCATAAATATGCATTATTTTTTCTATAGCTCATTGAATATGTATATCTGGCCATCCCATTCAGCATAAATTCCTGTCTTATTCTTTGGACCCTTGAAGTGTCTATTCCTAGTTTCTGGCTGGGGAGCTATGCTTCCCAGCTTGTCAGAACGGCCACCCTGCAGGCTATAATCCTTTATGAAAAACAAAGCTCTCCTTTCCATATTTATGAACCTTATTCTTCAGTTGACAAAACATAGCATATATCAACAGAAATTTGAAAAAAACAGTCAATCTGTGGATTCACTGGATTTTGTATGCACACAATCACACCATCTGCCAATAAAAGTATTCTTATTTATTTCCAATACTTCTACTTTGATTTTCTTCCTAACTATTTGGCTGAGAACTGCAGTAAAATACTAAATTGGGGTAGAAACAACATCATTGCCACATCTCCAAGCTTCACATTAGGTATATCTCCAAATATGTATTACCAAATAGGGGCTTAAGGGGTTCCCTTTTATTTCTATTGTTCTCAGCTTCTTCCCTCAAAAATCCAAAGTCTCTCTGAATTTTTAAGTTTTTAAATGTTTTCATCTGTTGTGATGATGGTATTATTTTTCTTCCATAATCTGTTCATGTGATTATTTTAAAACATTGTGATTTTAGAAAAATCCAACTAGCTTATGACATACCCTTTTTATGTATTTCTAGATTAGAGGTGCCAGTAGTTTAAGATTTTGGTATCTATGTTCATGACTGATATTGGCTTCATTTATTCCTTTATTAAATGCTTGAAATTTAAAGTGA

CPHX2

Exon 1

TCCTCCCGAGTAGCTGGGATTACAGGCATCCACCATCACGCCCAGCTAATTTTTGAACTTTTTGTGGAGACGGGGTTTCGCCATGTTGGCCAGGGTGGTCTCCAACTCCTGACCTCAGGTGACCCCCCACCTCAGCCTCCCAAAGTGCTGGGATTACAGGTGTGAGCTACCACGCCCTGCCAGAACTCAGTTTTTAAGGTTTCTCTGAGGTCCCTTTGACTAAAGGGGTCAGTCCATTGAGTTGTTTGGGGGACTTGGAATTTTATTTTTAGTTCTCACAAGAGTGTACAGAATCGCTGTTATATAGAATAAGTGTGTGGTGACAGGCATGGAATCAGCTGTCAGACGGCCAGGGTCGGAGAGCTGACCCTGGGCTCTGAACCATTCTACACCAAAGGTTCTTACCTGTAAAATGAGGATGAAAATGTGATATTAAAGAATTAAAGAAGAAAATTAATCTCAATGAATAGAAGATAGGATTTGAAAAG

Exon 2

TTGGAAAGGCCCTGGACTGGAGTTTCGACATGTCTTCCCAAG

Exon 3

CTTTCCCAGCTGAAGAGGATCATCATAATGAAGAAAGACAAACAAAGAAAAAAAGAAAAACAAAACACCGACATAAATTTTCTGAAGAATTATTGCAGGAACTTAAGGAAATATTTGGAGAGAACGGTTATCCTGATTTCACCACTAGGAAAACACTGGCCAACAAATTTGATTGTCCGGTTAACGTGATAAAC

Exon 4

AACTGGTTCCAGAACAATAGAGCCAGACTTCCACCGGAAGAAAGACAAAGAATATTTCTTACTTGGAAAAAACACGATTTCCCAGTCCAAGCCTGTCCATTTTTAAGCCTCCAGGAAACCCAGGCTGCAGCTTCCAACTATGCCACAGAGCAGAGTTTTTCCTGTGCCAAGAGGGCTCTGATGAGAAGACCTGGTTGCTCCCTTCTGGAGAAACAGAGGATTGCCTGTCAACAGATGGGCTACAATTGCTTCTCTTTGGAGAACCAAGAGACTCCCAGTCAACAGGTGGGCTCCATGTGCTCTTCTCTGGAGAAACAAGGGATTCCCAGTCAACAGGTGGGTTCCCAGTGCTCCTATCTGGTCGCAGGTACTGAAAAGCATCCAGGCTATGCTTTGGAGTATGGAGGTGACACAGGAAGTGAGCATTCTACTGCCTATCGTTTTCTCAGCTACAACTCTGCAGAATGCCTTCATCCTCCCCCATCTTCTGTGCCATATTTTCATGGAGAAAGGACTGAAACCAGGGAAAGCCAGCATGCAAGTCCCTTCCTTTTGGATTACGCTCAAGGTGCTTACGGGGTGAAGAAAGACCATTGTCTTTGCTCATTCTGTCTCTCACTGCTGCAAGAACAGCAGCAGAATGATTGGCAGTATCACCCACAGCAGCACCAACAGCCTCAGAATTACTCAGAGGGGATGATGTTGCAGGAACAGCTGCCAATGGACTCGGGTCCTTGGGATCTAGAGAAGCAGTGGCCCTCGGCTCAGTCACAGCTGCAGAGTCAACTGCCTCAGAATAATGGAAAGCCGTTGTGCTCTCAACTGCAGCACGTGCCTCCCCAAATAGCTGCCAACTCACCCCTGCTGCCTCTGGGGCAAGATATGCAGGTAGGGGCTTCAAGCAACTCAGGACTCAAATGCAGCAGTTTTAGGTTGAGGGGTCTACACGGGCCTGCCACAGGAACCCAAGGATGCAGCTTTGCAAAGTATTGCTAAGTATCACAGAACCATAGCCACAGGTCAAGCCCACAGTAACCCACACAGCAGGCCAAAGCAAAGCCGTCTCCAGAAGCCTCCACATAAAAAATAAAAATAAAAATAAGTAAATAAATAAATAAAAATAAA
