# Supplementary figures and images for: Diversity of human and mouse homeobox gene expression in development and adult tissues
Source: BMC Dev Biol. 2016 Nov 3;16:40. doi: 10.1186/s12861-016-0140-y (PMC5094009; doi:10.1186/s12861-016-0140-y)

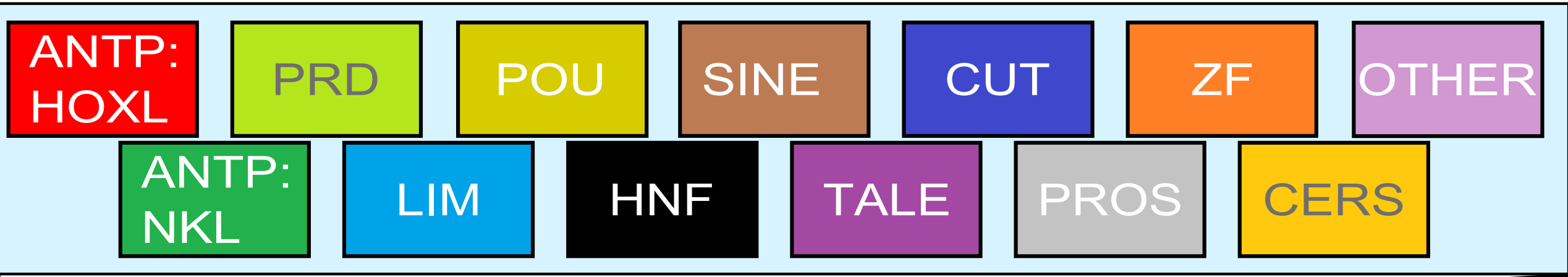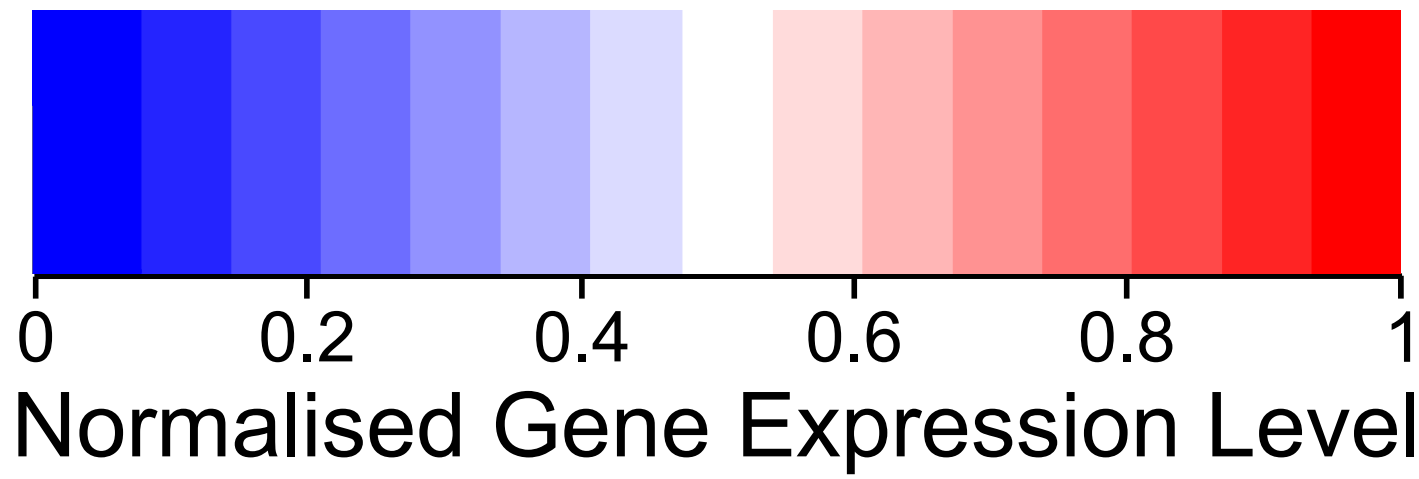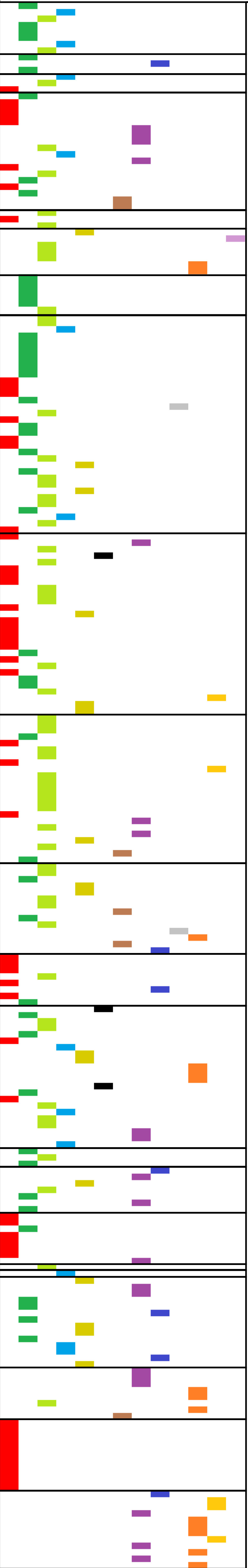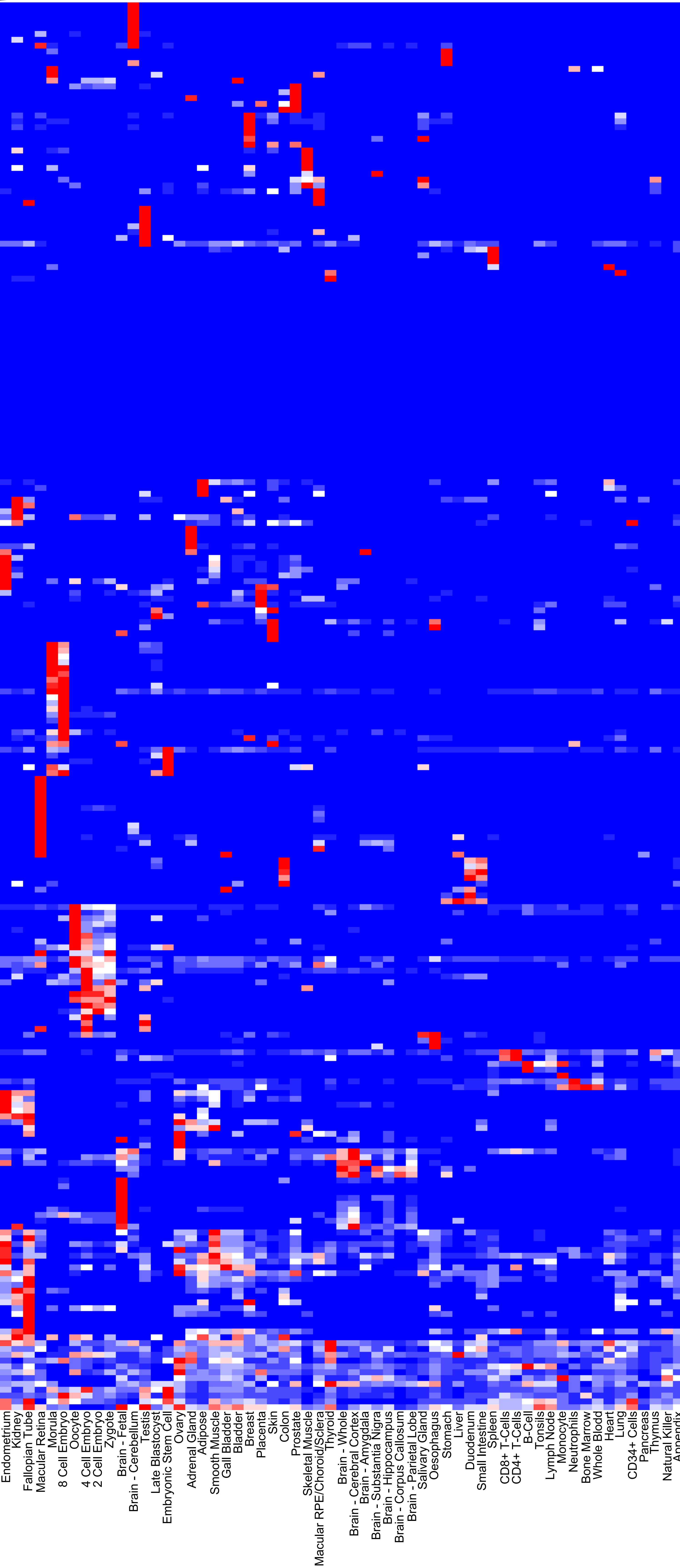

|          |  |
|----------|--|
| Table S3 |  |
| Table S4 |  |
| Table S5 |  |
| Table S6 |  |
| Table S7 |  |
| Table S8 |  |

Supplement: Additional file 4: Figure S2. — Heatmap showing human homeobox gene expression and gene names. Expression data for 242 homeobox loci across 59 human cell types and tissues clustered according to expression level after normalising individually to the maximal expression of each gene. Red high expression; blue low or zero expression. The horizontal coloured bars indicate the homeobox class for each gene. (PDF 271 kb) [file 12861_2016_140_MOESM4_ESM.pdf]

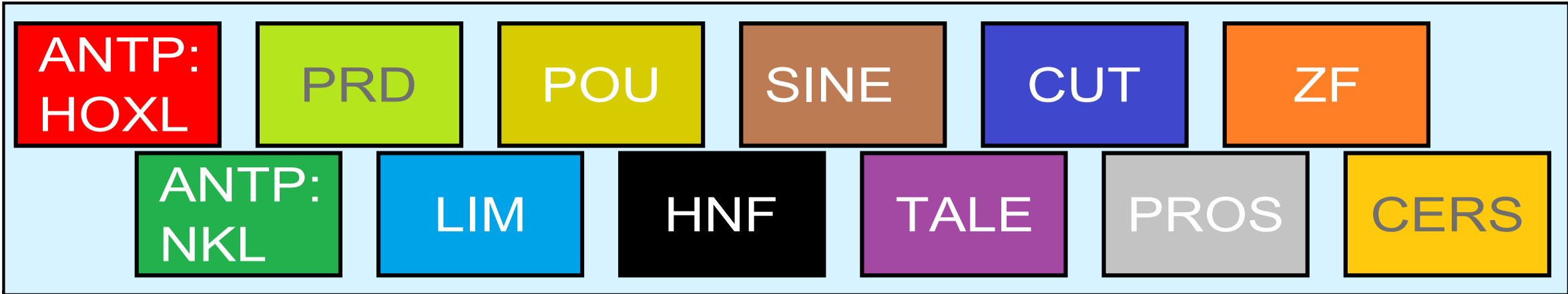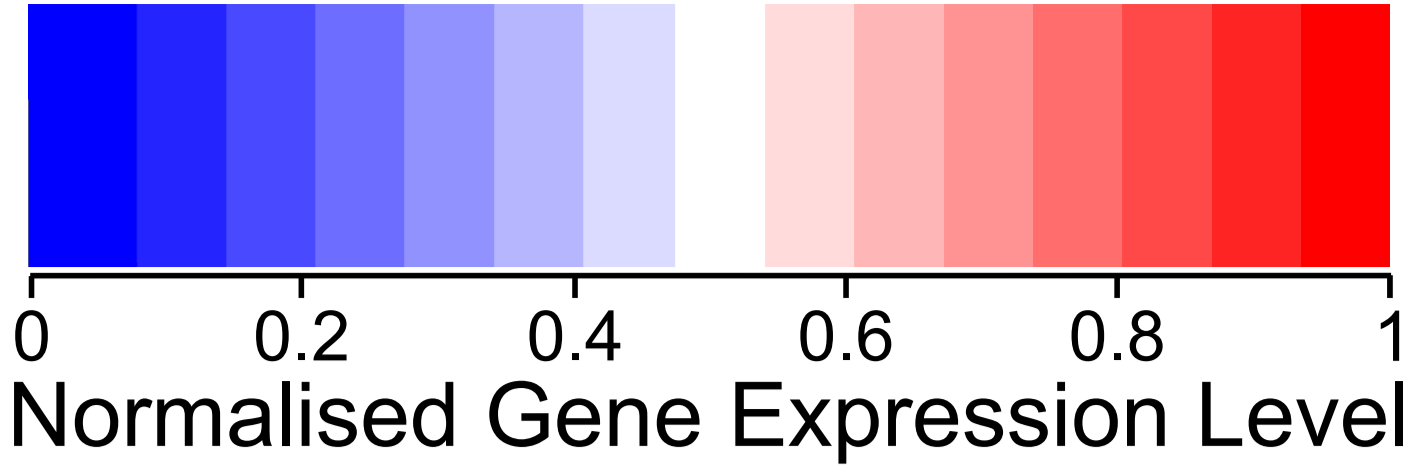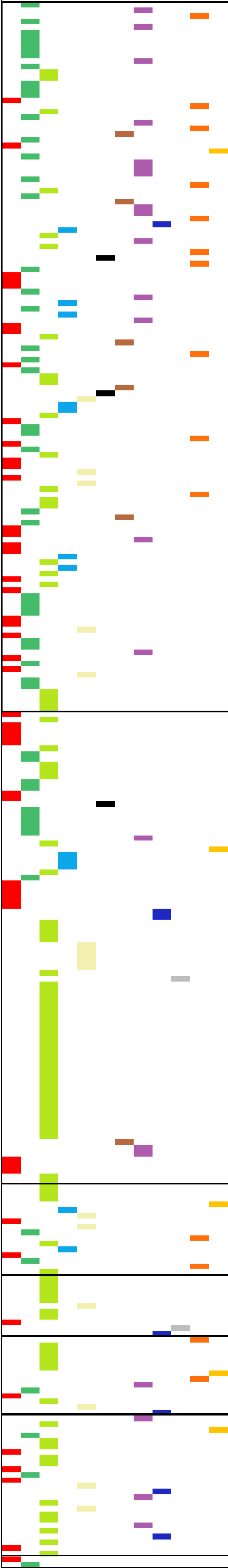

Phase 4

Phase 1+2

Phase 2

Phase 1

Phase 3

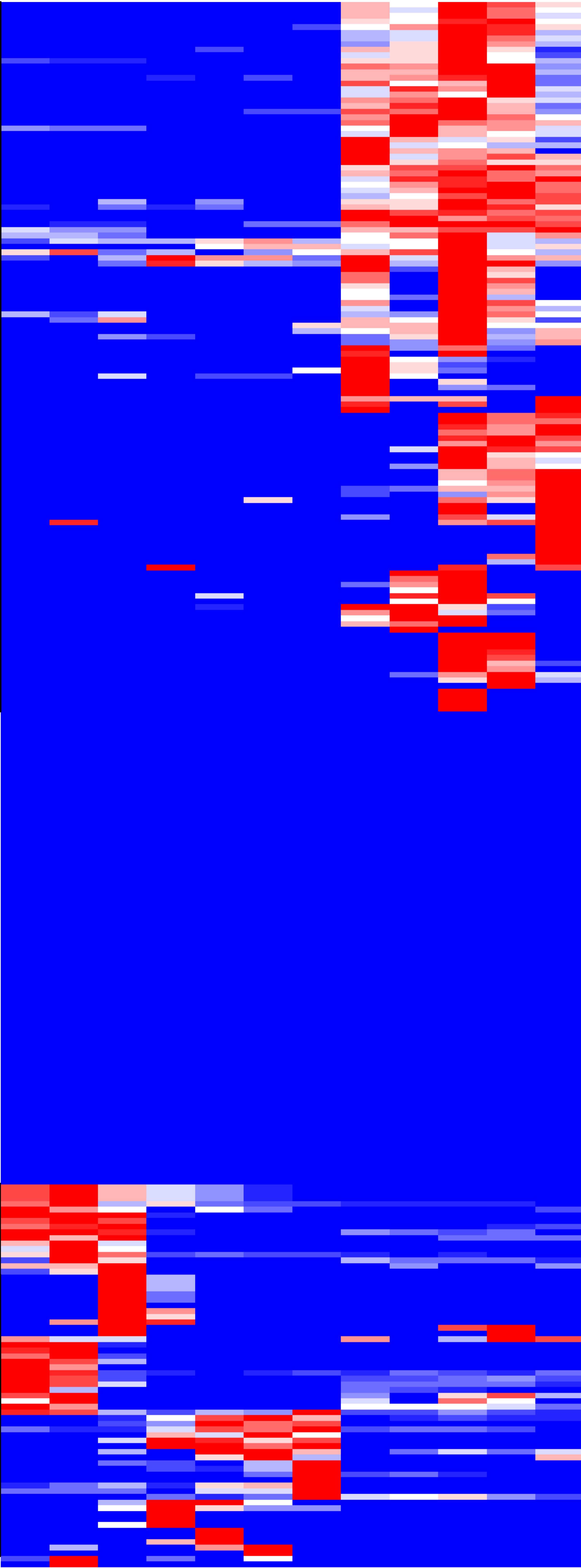

Oocyte  
Zygote  
2-Cell  
4-Cell  
8-Cell  
Morula  
Blastocyst  
e7.5  
e8.5  
e9.5  
e10.5  
e11.5

Supplement: Additional file 6: Figure S3. — Heatmap showing mouse homeobox gene expression in development and gene names. Expression data for 278 mouse homeobox loci across 12 embryonic stages clustered according to expression level after normalising individually to the maximal expression of each gene. The horizontal coloured bars indicate the homeobox class for each gene. (PDF 225 kb) [file 12861_2016_140_MOESM6_ESM.pdf]

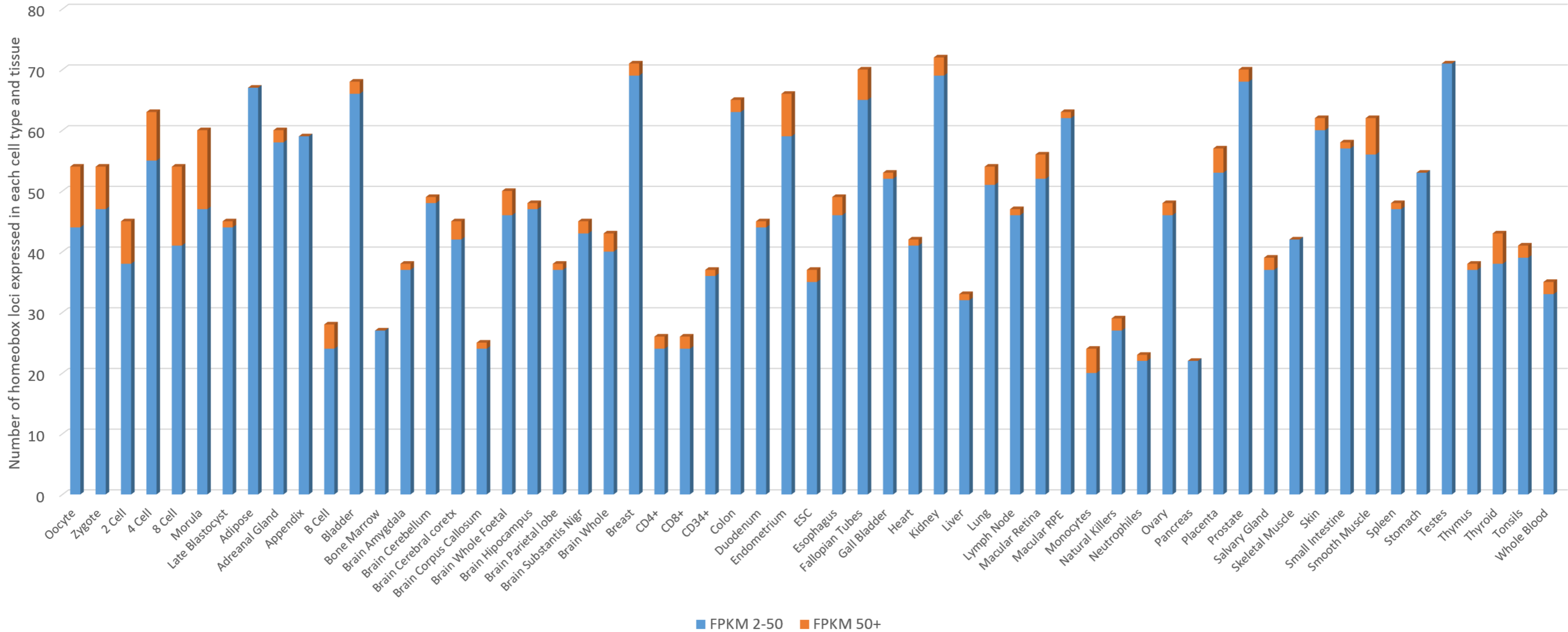

Supplement: Additional file 8: Figure S4. — The number of homeobox loci expressed in each of the assessed tissues and cell types. An FPKM of 2 was used as a cut off above which a loci was classed as expressed. Homeobox loci are separated by expression level for each cell type or tissue. (PDF 143 kb) [file 12861_2016_140_MOESM8_ESM.pdf]
